# Snacking Behaviour and Its Determinants among College-Going Students in Coastal South India

**DOI:** 10.1155/2018/6785741

**Published:** 2018-04-18

**Authors:** Prasanna Mithra, Bhaskaran Unnikrishnan, Rekha Thapar, Nithin Kumar, Sharana Hegde, Anjali Mangaldas Kamat, Vaman Kulkarni, Ramesh Holla, B. B. Darshan, Kanchan Tanuj, Vasudev Guddattu, Avinash Kumar

**Affiliations:** ^1^Kasturba Medical College, Manipal Academy of Higher Education, Mangaluru, Karnataka, India; ^2^Georgetown University Hospital, Washington Hospital Center, Washington, DC, USA; ^3^All India Institute of Medical Sciences, Basni Industrial Area, Phase-2, Jodhpur 342005, Rajasthan, India; ^4^Prasanna School of Public Health, Manipal Academy of Higher Education, Manipal, Karnataka, India

## Abstract

**Background:**

Consumption of snacks in between the regular meals is a poor snacking behaviour. It is an established risk factor for several lifestyle-related disorders and has long-term effects among the younger individuals.

**Objectives:**

To study the snacking behaviour and to assess their determinants among college-going students.

**Methods:**

This cross-sectional study was conducted among 865 college-going students in Mangaluru. Data were collected using a pretested pro forma that was coded and analyzed using Statistical Package for Social Sciences (SPSS) version 11.5. The chi-square test and random-effect logistic regression analyses were used.

**Results:**

Overall, there were 52.4% females and 47.6% males, and 76.8% of them were aged <20 years. More than half of the participants (54.3%) had the habit of snacking in between regular meals. Among them, a large proportion (78.7%) did not have any specific timings for snacking. Also, 51.1% of the students were snacking while watching TV, and 31.9% of them snacked while studying. Breakfast was the most commonly skipped meal (26.2%); of those missing the breakfast regularly, 123 (71.9%) had poor snacking behaviour. A significantly larger proportion of males had a higher frequency of snacking per day (69.3% versus 57.2%, *p* < 0.0001) and consumed aerated drinks more frequently (22.6% versus 15.8%, *p*=0.011), skipped meals more often (58.6% versus 50.6%, *p*=0.022), and preferred adding fruits in snacks (78.1% versus 69.4%, *p*=0.005). Snacking frequency was proportionately higher among students of private colleges (73.6%) than that in the government colleges (55.1%). Participants from nonscience stream, nonvegetarians, and those with a tendency to skip the regular meals had significantly higher levels of poor snacking behaviour.

**Conclusions:**

The study population had a high level of poor snacking behaviour. Appropriate measures are needed among younger people to follow fixed eating patterns and avoid skipping of regular meals.

## 1. Introduction

Snacking refers to the consumption of food and drinks including items such as chips, chocolates, and soft drinks. Studies from across the globe have shown high rates of snacking among young people, especially school-going students [1]. Consumption of snacks in between the regular meals amounts to poor snacking behaviour [1, 2]. Irregular snacking behaviour poses extreme risks to the health of children and adolescents including cardiovascular, neurological, and metabolic complications [2]. The extent of harmful effects increases with an early age of onset of irregular snacking behaviour [2].

Irregular eating habits are attributed to various factors like peer influence, parental behaviour, and influence of western food. Irregular snacking may even lead to skipping of regular meals and increased frequencies of snacking in between [2, 3]. In developed countries, a high percentage of children and college students have reported skipping of meals and increased snacking behaviour [3]. The regular breakfast which is the first nutritious meal of the day consumed on a regular basis helps in maintaining the positive health. Skipping breakfast leads to adverse health outcomes and also increases the chances of poor snacking behaviour [3]. Similar behaviour is observed among the children and adolescents of developing countries. India is also facing this transition, and more college students and adolescents are adopting western dietary styles along with the snacking behaviour. According to a previous research, very high proportion (62.1%) of adolescents had the habit of snacking in between meals [4]. Among several influencing and determining factors of adolescent snacking, parental behaviour and family status play important roles. Unhealthy snacking and skipping meals tend to be more common among adolescents from families where both parents are working [5]. Stress is also considered as an important factor which tends to influence snacking and eating patterns among young individuals [6].

Despite the potential link between intake of snacks and obesity and the reportedly high prevalence of snacking among adolescents, the snacking patterns of adolescents have not been extensively examined. Little is known about the context of snacking in adolescents or how snacking may influence other dietary habits, like skipping meals. This study was conducted among college-going students to assess the level of poor snacking behaviour, snacking patterns, and their lifestyle factors contributing to the pattern of food consumption, along with sociodemographic correlates.

## 2. Methods

This cross-sectional study was conducted among students pursuing graduation in different colleges of the Mangaluru city in Southern India between October 2013 and July 2016. Mangaluru is one of the port cities with a population of 398,745. It is a rapidly developing city with a high literacy rate [7]. The sample size was calculated as 865, assuming 50% of the students having poor snacking pattern (because of lack of previous studies reflecting the level of poor snacking behaviour), 10% relative precision, and 95% confidence interval (*α* error of 05%), adding 10% nonresponse error, considering a design effect of 2, and using the formula *n*={*z* ^2^ *p*(1 − *p*)/*d* ^2^}^∗^ design effect [8], where *p*=0.5, *z*=1.96 for 95% confidence interval, and “*d*” at 10% [9].

The study was approved by the Institutional Ethics Committee (IEC) of Kasturba Medical College (Manipal Academy of Higher Education), Mangaluru, India. Following this, the list of degree colleges was obtained from the District Education Authorities. The multistage sampling technique was used to select the study subjects. The list of colleges was stratified as government and private colleges which reflected the socioeconomic status of the study participants, wherein those from lower socioeconomic status mainly depended on the government run colleges. Here, the classes within the colleges were taken as the units of the study. Owing to the similarity of students within a class as a group, they were considered as clusters. Among 126 degree colleges, 6 were selected using stratified random sampling to get an equal number of colleges from each strata (3 government and 3 private), and students were selected from each of those colleges using cluster sampling. The colleges were visited on a preinformed date after seeking permission from the Heads of each of the selected colleges. The students were explained about the study, following which a written informed consent was obtained, and they were called in to participate in the study. For those subjects aged less than 18 years, a written assent was taken and their parents' written consent was taken through them, after which the face-to-face interview was conducted for each of the study participants. Those students who were not willing to participate in the study, remaining absent despite 2 visits to the college, and the ones suffering from serious medical illness were excluded from the study.

The data were collected using a prepilot-tested, content-validated (by the senior researchers and experts in the field of young adult nutrition), and semistructured pro forma that included questions on the diet and snacking patterns of the study participants. Consumption of food and drinks including soft drinks, chips, chocolates, and other fried items was considered as snacking, and consuming such snacks between regular meals for over >3 times a week was considered as poor snacking behaviour. The questions included their predominant diet (vegetarian or nonvegetarian), habit of having snacks in between regular meals, the frequency and time of snacking, preferred snacks, activity along with which snacking happened more frequently, reasons for snacking, tendency to miss regular meals, and their parents missing their regular meals. Any habit of consuming snacks in between regular meals was considered as poor snacking behaviour for the analysis.

The collected data were coded and entered into Statistical Package for Social Sciences (SPSS version 11.5 Inc., Chicago, IL, USA). The results were expressed as proportions. The chi-square test was used to compare the differences across the groups. Mixed-effect logistic regression analysis with deviance was carried out using STATA data analysis and statistical software (StataCorp LP) version 13, with poor snacking behaviour among the study participants as the outcome variable, against which the other variables were compared (available here). For odds ratios, all the other covariates used in the regression model were adjusted. *p* value <0.05 was considered as statistically significant.

## 3. Results

The study population included 865 college-going students. To reach the desired sample size, 900 eligible students had to be approached and the attrition rate (nonresponse rate) was 3.8%.  Table 1 describes the demographic details of the study population. Overall, the study participants comprised 453 (52.4%) females and 412 (47.6%) males. The participants were pursuing graduation courses in various disciplines, and most of the students were aged less than 20 years (76.8%).

More than half of the participants (54.3%) had the habit of snacking in between regular meals.  Table 2 depicts the snacking behaviour of the study participants. Among the participants who had the habit of snacking in between the regular meals, a large number (78.7%) did not have any specific timings for snacking. Breakfast was the most commonly skipped meal (26.2%). Also, 51.1% of the students were snacking while watching TV.

The genderwise distribution of general snacking behaviour is described in Table 3. Males had a higher frequency of snacking per day (69.3% versus 57.2%, *p* < 0.0001) and consumed aerated drinks more frequently (22.6% versus 15.8%, *p*=0.011). A significantly higher proportion of females preferred snacks over fixed meals (53.8% versus 46.4%, *p*=0.035).

Snacking frequency was proportionately higher among students of private colleges (73.6%) than that in the government colleges (55.1%). In contrast, 58.5% of the students from the government colleges preferred snacks over fixed meals compared to the students from private colleges (39.5%) (*p* < 0.05). The snacking behaviour of the study population with respect to the type of management of schools in which they studied has been depicted in Table 4. Snacking frequency ≥ 2 per day, consumption of aerated drinks regularly, and preference of taste over the nutritive value of the food item were significantly higher among the students from private colleges (*p* < 0.05).

Table 5 shows unadjusted and adjusted odds ratios (ORs) for the covariates of poor snacking behaviour among the study subjects. Tendency to skip meals had the highest adjusted OR (2.94 (95% CI: 2.17–4.00)), followed by the predominant diet (nonvegetarian diet 1.74 (95% CI: 1.29–2.35)) and subject specialty/stream in which they studied (nonscience 1.59 (95% CI: 1.07–2.36)). All the covariates showed statistically significant (*p* < 0.05) association with snacking behaviour in the study group.

## 4. Discussion

Snacking behaviour varies across the different regions of the globe. With the rapid development and the changing lifestyles of adolescents, the eating habits have also been changing. These are reflected in the current study findings. There have been studies from developed countries regarding snacking and eating behaviour of school- and college-going students and adolescents in general. Cros et al. from the United States of America reported that 87-88% of adolescents aged 12–18 years consumed at least one snack per day with snacks contributing approximately 25% of their daily energy intake [10]. A study by Anderson et al. in Scotland reported an average of 5.5 eating occasions per day (2.7 main meals and 2.8 snacks), these eating occasions being concentrated towards the end of the day [11]. A similar study by Marques et al. showed that Portuguese youth (aged 5–15 years) consumed 1.5 snacks per day [12]. In Asian countries, snacking rates among youth (aged 2–19 years) are more variable. For example, in the Philippines, Russia, and China, 86%, 71%, and 10% of youth consume at least one snack on a daily basis, with snacks providing 18%, 16%, and 1% of their total daily energy, respectively [13]. Our study showed that 54.3% of the subjects consumed at least one type of snack beyond the timings of regular food intake for >3 times a week. Out of these, 78.7% did not have any specific timing patterns for snacking.

Our study observed a higher proportion of students from government managed colleges having poor snacking behaviour as compared to those from private Colleges. Commonly, those from lower socioeconomic class families depend on the government run colleges, and this selection in our study reflects the indirect way used to assess the socioeconomic status of the study participants. However, the current snacking behaviour could be explained by the fact that the snacks are available easily and more economically as compared to regular food items. Thus, the easier availability can trigger the snacking behaviour among those from lower socioeconomic background. Another influencing factor could be television viewing, which our study subjects stated as one of their most frequent activities while snacking. The programmes and advertisements in the television in turn can promote the use of snacks and enhance the poor snacking behaviour. Savige et al. from Australia found that the most common contexts for snacking among adolescents were after school (4.6 times per week), while watching TV (3.5 times per week), and while hanging out with friends (2.4 times per week) [14]. Another study showed that non-Hispanic adolescents showed stronger association between television exposure and cravings for sweet snacks, salty snacks, and sweetened drinks which was similar to our findings. They also found that being Hispanic was associated with stronger associations between phone messaging and cravings for sweet snacks, salty snacks, and sweetened drinks and that males showed stronger associations between video game use and salty snack cravings [15]. Similarly, Falbe et al. found that the increase in screen time (i.e., television, electronic games, digital versatile discs (DVDs)/videos, and total screen time) was associated with increased consumption of foods and beverages of low nutritional quality and decreased consumption of fruits and vegetables [16]. Our study observations are similar to other studies as snacking was associated with watching TV in more than half of the study participants. Grenard et al. found that watching TV was associated with consuming sweet snacks but not with salty snacks or sweet drinks [17]. Studies by Santaliestra-Pasías et al. [18], Pearson et al. [19], Ouwens et al. [20], and Niven et al. [21] also found that watching TV was associated with increased snacking. Our study showed that, among the students who snack, a majority of the students (51.1%) snack while watching TV, as compared to while studying or while hanging out with friends concurrent with the above studies. Cros et al. found that children and adolescents select snacks based on taste over nutrition. They more often choose salty, crunchy foods as snacks over healthier alternatives [10]. Ming et al. found that, out of a total of 3508 students, 19.9% (*n*=699) of them skipped at least one meal a day. The most frequently missed meal was breakfast (12.6%), followed by lunch (6.7%) and dinner (4.4%) [22].

Another study also had similar findings with breakfast being the most commonly skipped meal [23]. Our study showed that 37.5% of a total of 865 students skip meals, among which breakfast was the most commonly skipped meal (37.9%, *n*=324), followed by lunch and then dinner. This has direct implications on the long-term health status of young individuals because the importance of timely breakfast and not skipping it has already been proved to be beneficial for health [3].

The association between gender and snacking behaviour has been variable in previous studies from different regions. More frequent snacking has been reported among boys than girls [24, 25] and vice versa [26], while a few studies did not observe any gender differences in snacking behaviour at all [10, 27]. Anding et al. found that males reported an average of 2.6 snacks per day compared to the 1.9 snacks per day reported by females (*p* > 0.05) [28]. Our study observations are similar in this regard and showed that more number of males snack frequently (more than twice a day) compared to females. On comparing male and female snacking behaviour, we observed that females more often tend to replace their meals with snacks (53.8%) and had a tendency to skip meals (58.6%). Males tend to snack more of oily snacks and aerated drinks as compared to females who include more fruits in their snacks. The pattern of skipping meals varied with gender and area of inhabitance. Females were more likely than males to skip breakfast and lunch. Similarly, adolescents from metropolitan areas were more likely than their peers from nonmetropolitan areas to skip breakfast. Our observations are similar to previous studies that have reported a higher frequency of skipping of breakfast among female adolescents [29, 30].

Also, the study population in the computer applications and science stream had lower levels of poor snacking behaviour as compared to those in nonscience stream. There could be multiple factors responsible for this difference. The students with science background are taught and trained about various aspects of diet and its biological concepts as part of the curricula. Thus, the knowledge about the snacking behaviour among the subjects from computer applications and science background could translate into better behaviour.

Overall, the strategies, policies, and plans addressing the snacking behaviours have to be based on the optimum understanding along with the analyses of complex outcomes of snacking behaviours on their weight [31]. This study is one of its kind taking into account the educational stream along with other covariates influencing the snacking pattern of the young individuals. Limitations of the current study could be that factors such as stress and emotion that are likely to influence snacking behaviour were not taken into consideration.

## 5. Conclusions

Our study reports poor snacking behaviour among the younger generation of the region. The various aspects of snacking behaviour varied across gender, stream of study, and socioeconomic status. The tendency to skip regular meals and nonvegetarian diet were associated with the poor snacking behaviour among the study population.

## Figures and Tables

**Table 1:** Demographic details of the study population (*n*=865).

Characteristic		Number (%)
Age group (years)	<20	664 (76.8)
	≥20	201 (23.2)

Gender	Male	412 (47.6)
	Female	453 (52.4)

Subject specialty/stream	Computer applications and science students	146 (16.9)
	Nonscience students	719 (83.1)

School management	Government	490 (56.6)
	Private	375 (43.4)

Predominant diet	Vegetarian	390 (45.1)
	Nonvegetarian	475 (54.9)

**Table 2:** Snacking behaviour of the study population.

Characteristic		Number (%)
Snacking in between the regular meals (*n*=865)	Yes	470 (54.3)
	No	395 (45.7)

Time of snacking (*n*=470)	Not specific	370 (78.7)
	Mid-morning	029 (06.2)
	Before dinner	044 (09.4)
	After dinner	023 (04.9)

Activity during which snacking is most common (*n*=470)	Television viewing	240 (51.1)
	Studying	150 (31.9)
	Hanging out	031 (06.6)
	No specific activity	049 (10.4)

Meal that was most commonly skipped among subjects with poor snacking behaviour (*n*=470)	Breakfast	123 (26.2)
	Lunch	104 (22.1)
	Dinner	070 (14.9)
	Anyone/more than one	027 (05.7)
	No response	146 (31.1)

Meal that was commonly skipped by subjects without poor snacking behaviour (*n*=395)	Breakfast	048 (12.2)
	Lunch	044 (11.1)
	Dinner	044 (11.1)
	Anyone/more than one	010 (02.5)
	No response	249 (63.0)

Reasons for snacking (*n*=470)	Snacks are tastier	221 (47.0)
	To lose weight	036 (07.7)
	Snacks are convenient	032 (06.8)
	No specific reason	181 (38.5)

**Table 3:** Snacking behaviour of the study population according to the gender (*n*=865).

Characteristic	Number of males/total (%)	Number of females/total (%)	*p* value
Snacking in between meals	228/412 (56.4)	242/453 (54.7)	0.618
Snacking frequency > twice a day (irrespective of the timings)	276/398 (69.3)	254/444 (57.2)	0.0001^∗^
Consumption of oily food items > 4 times a week	306/398 (76.8)	315/444 (70.9)	0.051
Consumption of aerated drinks > 4 times a week	90/398 (22.6)	70/444 (15.8)	0.011^∗^
Preference of snacks to fixed meals	183/394 (46.4)	235/437 (53.8)	0.035^∗^
Tendency to skip meals	200/395 (50.6)	249/425 (58.6)	0.022^∗^
Inclusion of fruits in snacks	268/386 (69.4)	338/433 (78.1)	0.005^∗^
Taste over nutrition	263/390 (67.4)	280/425 (68.2)	0.807

^∗^ *p* value significant at 0.05 cutoff level.

**Table 4:** Snacking behaviour of the study population according to the type of school management.

Characteristic	Number of government college students/total (%)	Number of private college students/total (%)	*p* value
Snacking in between meals	277/480 (57.7)	193/364 (53.0)	0.175
Snacking frequency > twice a day (irrespective of the timings)	270/490 (55.1)	276/375 (73.6)	<0.0001^∗^
Consumption of oily food items > 4 times a week	364/490 (74.3)	274/375 (73.1)	0.686
Consumption of aerated drinks > 4 times a week	044/490 (09.0)	118/375 (31.5)	<0.0001^∗^
Preference of snacks to fixed meals	282/482 (58.5)	147/372 (39.5)	<0.0001^∗^
Tendency to skip meals	284/470 (60.4)	180/371 (48.5)	0.001^∗^
Inclusion of fruits	349/475 (73.5)	275/367 (74.9)	0.632
Taste over nutrition	285/461 (61.8)	278/372 (74.7)	<0.0001^∗^

^∗^ *p* value significant at 0.05 cutoff level.

**Table 5:** Determinants of poor snacking behaviour among the study participants based on random-effect logistic regression analysis (*n*=865).

Characteristics		Poor snacking behaviour (*n*=470), number (%)	Unadjusted OR (95% CI)	Adjusted OR (95% CI)
Gender	Female (*n*=453)	242 (53.4)	—	—
	Male (*n*=412)	228 (55.3)	1.12 (0.84–1.49)	1.07 (0.79–1.45)

Age group (years)	<20 (*n*=664)	343 (51.7)	—	—
	≥20 (*n*=201)	127 (63.2)	1.58 (1.09–2.27)	1.54 (1.05–2.27)

Subject/stream	Computer applications and science (*n*=146)	057 (39.0)	—	—
	Nonscience (*n*=719)	413 (57.4)	1.87 (1.27–2.75)	1.51 (1.01–2.25)^∗^

School management	Government (*n*=490)	277 (56.5)	1.09 (0.21–5.74)	1.16 (0.29–4.62)
	Private (*n*=375)	193 (51.5)	—	—

Predominant diet	Vegetarian (*n*=390)	188 (48.2)	—	—
	Nonvegetarian (*n*=475)	282 (59.4)	1.73 (1.30–2.31)	1.74 (1.29–2.35)^∗^

Usual tendency to skip any meal	Yes (*n*=464)	324 (69.8)	3.12 (2.27–4.16)	2.94 (2.17–4.00)^∗∗^
	No (*n*=401)	146 (36.4)	—	—

^∗^ *p* value significant at 0.05 cutoff level; ^∗∗^ *p* < 0.0001; deviance = 74.32.

## Additional Points

*Recommendations*. There is a need to increase the awareness among younger generation towards the health impact of snacking, poor snacking behaviour, and importance of following the regular meal pattern. Also, some curriculum-oriented health education needs to be implemented for younger people in the institutions, highlighting the importance of snacking behaviour in health.

## Supplementary Materials

Supplementary Materials The data file includes the variables incorporated in this study.
